# Modulation of Spatial Attentional Allocation by Computer-Based Cognitive Training during Lacrosse Shooting Performance

**DOI:** 10.3389/fpsyg.2017.02271

**Published:** 2018-01-04

**Authors:** Takahiro Hirao, Hiroaki Masaki

**Affiliations:** ^1^Graduate School of Sport Sciences, Waseda University, Saitama, Japan; ^2^Faculty of Sport Sciences, Waseda University, Saitama, Japan

**Keywords:** reversed Simon effect, Simon task, stimulus–response compatibility, lacrosse, computer-based sport training

## Abstract

It has been reported that repetitive execution of a stimulus–response compatibility (SRC) task attenuates the interference effect of a choice reaction time task, known as a Simon task. We investigated whether attentional control, enhanced by repetitive execution of an SRC task, would reduce the interference effect of a Simon task and could be transferred to lacrosse shooting skills, increasing the likelihood that players would shoot in the direction opposite to the goalie’s initial movement. Female lacrosse players who were matched in terms of age, handedness score, competitive lacrosse playing experience, and playing position, were allocated to the SRC task group (*n* = 15) or the 2-back training group (*n* = 14). Participants underwent 10 sessions of 180 trials of a computer-based version of either a Type 2 SRC task or the 2-back task, within four consecutive weeks. Eight practice trials were completed prior to the execution of each task in every training session, during which feedback was provided to confirm accurate mapping between the stimulus and response. Before and after the training phase, both the magnitude of the Simon effect and the lacrosse shooting performance were assessed. After participating in computer-based cognitive training, players did indeed increase the number of shots toward the direction opposite to that of the movement of the goalie. In conclusion, these findings indicate that computer-based cognitive training is beneficial for improving the shooting ability of lacrosse players.

## Introduction

Lacrosse is an ancient sport that originated in North America. Many studies of lacrosse have focused on the physical aspects of lacrosse players, such as strength and conditioning (e.g., Gutowski and Rosene, 2011), or kinematic variables that contribute to ball velocity and the accuracy of lacrosse shots (e.g., Macaulay et al., 2016). However, relatively few studies have investigated the cognitive aspects of lacrosse, such as attentional control and decision-making for an appropriate response.

In lacrosse, shooting in the direction opposite to that of the goalie’s movement can be an effective strategy for scoring, because the goal-width in lacrosse is quite narrow (1.83 m × 1.83 m). The shooter needs to observe the movement of the goalie carefully and shoot in the direction opposite of the goalie’s movement as quickly as possible after the goalie starts to move. However, shooting in the opposite direction of the goalie’s movement is not easy, due to the spatial incompatibility of the direction of the goalie’s movement and the spot the shooter should target.

It has been reported that processing the body movement of another person is crucial in social communication and is an automatic process (Langton and Bruce, 2000). In lacrosse, irrespective of the shooter’s intention, the shooter’s attention is automatically allocated to the movement direction of the goalie, and thus the related response preparation is also automatically initiated. In many sports, a common technique that takes advantage of this automatic allocation of attention is the feint; sometimes referred to as a “fake” or “deke” (Kunde et al., 2011). In terms of lacrosse shooting performance, this automatic attentional mechanism could affect the attention allocation and response preparation of the shooter, resulting in sub-optimal shot execution.

The purpose of this study was to determine if the attention-allocation strategy induced by the repetition of computer-based training could transfer to the attentional control ability of the lacrosse shooter’s performance. It has been reported that performing computer-based tasks or games affects the attentional system. Green and Bavelier (2003) determined that playing action video games altered visuo-attentional processing through a series of five experiments. Playing action video games for 1 h per day for 10 days improved visuo-attentional abilities as assessed by psychological tasks (see Experiment 5 of Green and Bavelier, 2003). Their findings implied that short-term intervention with computer-based training may alter visuo-attentional processing.

Previous sport-related studies have indicated that attentional control acquired through computer-based training can transfer to performance in the actual game situation. Fery and Ponserre (2001) showed that novice golfers improved their putting skills through playing a computer-based golf-simulated task. In their study, participants learned an adequate force control for putting from computer-based training and a positive transfer of training to the actual putting skill was shown. Computer-based training was also used as an attractive training tool to improve the motor skills of elderly people. Intervention using computer-based training helped to improve gait performance (Pichierri et al., 2012) and postural control in elderly adults (Pluchino et al., 2012; Donath et al., 2016). However, no study has yet focused on training-induced modulation of the attentional allocation in sport-related research. Adequate attentional allocation could lead to a better performance in sports games (e.g., Vickers, 2011). Therefore, modulating the attention-allocation strategy could be meaningful for athletes playing competitive sports.

In laboratory-based psychological research, the attentional mechanism underlying spatial incompatibility between the stimulus location and reaction has been investigated using a choice reaction time task known as a Simon task (Simon, 1969). In the typical Simon task, a fixation cross is shown at the beginning of the task. Thereafter, one of two types of stimuli (e.g., a green or red circle) is presented to the left or the right of the fixation point. The participant’s task is to respond with their left hand to the green circle and with their right hand to the red circle, irrespective of the location of the stimulus presentation. When the response hand corresponds to the side on which the stimulus is presented (i.e., when the green circle is presented on the left side or the red circle is presented on the right side), the reaction time is faster than when the response hand does not correspond to the stimulus location. The spatial correspondence between the stimulus and response thus affects the reaction time to the stimulus.

This phenomenon, known as the Simon effect, is thought to be caused by the automatic attentional shift to the task-irrelevant location of the stimulus presentation (Notebaert et al., 2001); this initiates the corresponding automatic response preparation (Masaki et al., 2000). The conflict between automatic brain activation during the processing of the spatial information, and the conditional activation related to response selection, could cause a delay in the reaction to an incompatible stimulus at the level of response selection. Thus, the Simon effect may be produced at both the perceptual and the response-selection stages. This hypothesis was confirmed by studies that recorded event-related brain potentials, including both the lateralized readiness potential and P300 (Valle-Inclán, 1996; Masaki et al., 2000).

The Simon effect is a robust phenomenon and has been replicated in numerous studies (see review by Lu and Proctor, 1995). However, after much repetition of a simple stimulus–response compatibility (SRC) task in which participants respond to an eccentrically presented stimulus with a spatially incompatible response button (i.e., pressing the right button to respond to a stimulus located on the left side and vice versa), the Simon effect is reversed, resulting in shorter reaction times in incompatible trials than in compatible trials (Proctor and Lu, 1999). For the Simon task, the stimulus type (e.g., color or letter) is task-relevant, whereas the stimulus location is task-irrelevant. However, in the above-mentioned simple SRC task, the stimulus location is relevant, whereas the stimulus type is irrelevant for the incompatibility stimulus (i.e., irrespective of the stimulus type, participants must react to the opposite side of the spatial location of the stimulus).

Therefore, the Simon task and the SRC task can be distinguished by a taxonomy, as proposed by Kornblum (1992). According to this taxonomy, individual SRC tasks can be classified into one of eight different ensembles in terms of the dimensional overlap and dimensional relevance of the stimulus and response, depending on whether the relevant and irrelevant stimulus dimensions, or the stimulus and response dimensions, overlap conceptually. For the Simon task, there is an overlap between the dimensions of an irrelevant stimulus (i.e., location) and the response (i.e., left/right), but there is no overlap between the dimensions of a relevant stimulus (i.e., color or letter) and response or between the dimensions of relevant and irrelevant stimuli. This taxonomy designates this kind of task as a Type 3 ensemble. For the above-mentioned SRC task, there is an overlap between the relevant stimulus (location) and response dimensions (left/right), but such overlap is absent for the irrelevant stimulus (letter) and response dimensions, and for the relevant and irrelevant stimulus dimensions. The taxonomy describes an SRC task, such as that of Proctor and Lu (1999), as a Type 2 ensemble. Thus, the taxonomy clearly ascribes the Simon and SRC tasks to distinct types.

It has been reported that repeated exposure to an SRC task with incompatible spatial mapping could affect response performance for the Simon task (Proctor and Lu, 1999; Tagliabue et al., 2000, Tagliabue et al., 2002). A reversal of the Simon effect was observed after participants performed more than 1800 trials of a Type 2 task. Extended practice of a Type 2 task with incompatible spatial mapping may therefore create a temporal association between the incompatible stimulus and its response (Proctor and Lu, 1999).

However, the amount of practice required may involve substantially fewer than 1800 trials. Tagliabue et al. (2000) showed that the magnitude of the Simon effect could be decreased after performing only 72 trials of a Type 2 task. Moreover, repetition of the Type 2 task reduced the Simon effect not only immediately after the execution of the spatially incompatible task, but also after delays of 1 day and 1 week (Tagliabue et al., 2000). This decreased Simon effect was considered to be the result of short-term memory association and consolidation of the incompatible spatial mapping response after repetition of the Type 2 task. The conditional short-term memory association that was created by the task-relevant instruction (i.e., the instruction to react to the opposite side of the stimulus presentation) altered the long-term memory association, which was influenced by the automatic attentional orienting mechanism of the oculomotor system (Tagliabue et al., 2000, Tagliabue et al., 2002). According to these studies, it is plausible that repetition of a Type 2 task can diminish the magnitude of the Simon effect, and perhaps even reverse it, for at least 1 week after execution of the SRC task.

We used the Type 2 task as a cognitive training tool to strengthen the temporal-memory association with the incompatible spatial mapping. According to Proctor and Lu (1999), repetition of the Type 2 task (i.e., 1800 trials) with an incompatible spatial mapping decreased the interference effect on the Simon task. Thus, repetitive exposure to the Type 2 task may result in reversal of the Simon effect, although the performance reached an asymptote due to a ceiling effect because of the simplicity of the Type 2 task. The repetitive practice of a Type 2 task could lead to a new association of the incompatible spatial mapping between the stimulus and its response. It has already been demonstrated that the state of the incompatible spatial mapping could transfer in the Simon task (i.e., leading to a reversed or attenuated Simon effect). However, no study to date has assessed the effect of the repetition of an SRC task in a real-life situation, such as playing sports.

The aim of the current study was to clarify the effect of repetition of a Type 2 task on lacrosse shooting performance. As repetition of a Type 2 task could decrease the Simon effect, it may be possible to allocate the athlete’s attention to the opposite side of the stimulus presentation, and thus prepare them for a response toward the opposite side. We hypothesized that lacrosse shooters who received repetitive SRC training would shoot more often in the direction opposite to the goalie’s movement than a control group who performed a 2-back memory task that does not involve spatial stimulus–response mapping. Moreover, we postulated that the magnitude of the decrease of the Simon effect induced by repetition of the Type 2 task would correlate with the increase in the number of shots in the direction opposite to the goalie’s movement.

## Materials and Methods

### Participants

Twenty-nine college-level female lacrosse players from Waseda University’s women’s lacrosse team, aged 19-23 years (*M* ± *SD*: 20.6 ± 1.1), volunteered to participate in this study. Hand preferences were assessed with the Edinburgh handedness inventory (Oldfield, 1971). Participants were assigned either to the SRC task group (i.e., the experimental group) or the 2-back task group (i.e., the control group). The control and experimental groups were matched based on pre-test shooting ability as well as on personal characteristics (i.e., age, competitive experience, Edinburgh handedness score, and lacrosse playing position), acquired through questionnaires, to minimize any preexisting differences in terms of lacrosse skill and other features between the two groups. The participants in the SRC task group (*n* = 15) and the 2-back training group (*n* = 14) were not significantly different in terms of age, handedness score, competitive playing experience of lacrosse, or position, *p*s > 0.05 (**Table 1**). This study was approved by the Ethics Committee of Waseda University, and informed consent was obtained from all participants.

**Table 1:** The characteristics of each group.

	*N*	Age (years)	Competitive experience (years)	Edinburgh handedness score	Playing position		
					
					AT	MD	DF
SRC group	15	20.3 ± 1.0	2.8 ± 1.8	81.1 ± 48.1	7	3	5
2-Back group	14	20.8 ± 1.1	3.0 ± 2.0	80.6 ± 24.2	5	4	5


*There were no significant differences between the two groups. AT, attacker; MD, midfielder; DF, defender.*

### Procedure Overview

**FIGURE 1:**
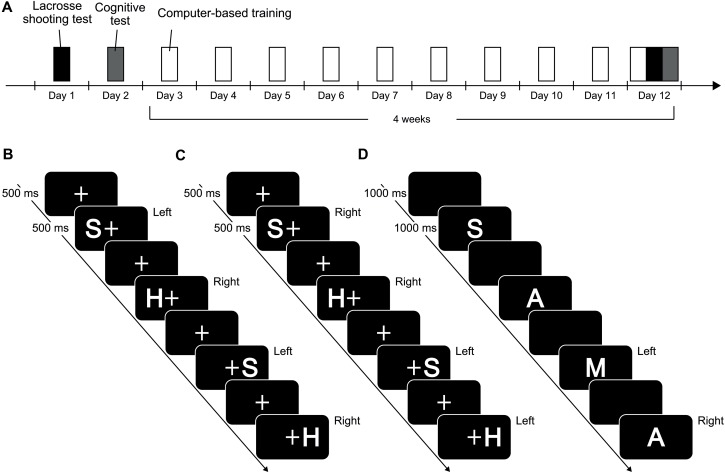
Sequences of stimulus and response events in the Simon task, Type 2 task, and 2-back task. The correct hand with which to react to each stimulus is depicted in the upper right section of each stimulus. **(A)** Schematic illustration of the design of the study. **(B)** Schematic illustration of the Simon task. Participants were asked to respond with their left or right index fingers based on which character (H or S) was presented. Participants had to press with their left finger for the character “S” and with their right finger for the character “H.” **(C)** Schematic illustration of the Type 2 task. In the Type 2 task, participants were asked to respond with their left or right index fingers according to the type of spatial information (left or right). Participants had to press with their left finger for right-sided presentation of characters, and with their right finger for left-side presentation of characters. **(D)** In the 2-back task, participants had to answer whether the current stimulus was identical to the stimulus that preceded it by two. When the same stimulus as that occurring “2-back” was presented, participants were asked to respond with their right index finger. If it was different, they were to respond with their left finger. Four types of characters were used (A, H, M, S).

All participants performed lacrosse shooting tests, cognitive tests, and underwent a training phase. In the training phase, a computer-based cognitive training task (i.e., either SRC or 2-back task) was conducted 10 times within four consecutive weeks. Before and after the training phase, shooting performance and cognitive skills were assessed in a lacrosse shooting test and a Simon task, respectively (**Figure 1A**). On the first day, each participant completed an informed consent form, a brief questionnaire, and the Edinburgh handedness inventory, after receiving an explanation of this experiment. After completing the questionnaires, participants performed the lacrosse shooting test. They were then assigned to either the SRC training group (the experimental group) or the 2-back task group (the control group) based on the pre-test lacrosse shooting and other personal characteristics (**Table 1**). On the second day, cognitive skills during the Simon task were evaluated. From the 3rd day to the 12th day, computer-based cognitive training was carried out. During the training phase, participants performed 180 trials of the computer-based task in a day. The training phase consisted of 10 sessions. Thus, participants performed a total of 1800 trials of the computer-based task during the training phase. In each training session, the experimenter gave the participant the same task instructions prior to the training, and reconfirmed that participants followed the instructions during execution of the computer-based tasks. According to Proctor and Lu (1999), performing a minimum of 1800 trials of the Type 2 task appears to be a necessary condition to establish the short-term-memory association of the incompatible spatial mapping. To meet this condition, participants were allowed to conduct a few sessions in a day when they did not keep up with the pre-required training schedule. The inter-session intervals did not differ between the SRC group and the 2-back group, *t*s(27) ≤ 1.4, *p*s ≥ 0.17. On the 12th day (i.e., the final session of the training phase), the 10th computer-based cognitive training session was followed by both the lacrosse shooting and cognitive tests. Due to a scheduling problem, one participant in the SRC group and one participant in the 2-back group conducted the lacrosse shooting test 2 and 3 days after the final training session, respectively. In addition, one participant in the SRC group and three participants in the 2-back group conducted the cognitive test several days (ranging from 2 to 6 days) after the 10th training session. All tests and training were conducted on the lacrosse playing field (i.e., the facility of the Waseda University). Because all computer-based tasks were conducted using a laptop computer before and after the lacrosse club activity, participants could perform computer-based tasks in a quiet environment. The monitor of the laptop was placed 1 m in front of the participant (maximum visual angles were 6.1° × 1.9° for the Simon and Type 2 task, and 1.9° × 1.9° for the 2-back task). In all computer-based tasks, “z” and “/” keys on the keyboard meeting a Japanese industrial standards layout were used to respond with a left and right index finger, respectively.

### Cognitive Test

All participants performed 180 trials of the Simon task before and after the training phase to examine the degree to which they demonstrated the Simon effect. In our study, two letters (“H” or “S”) were used as visual stimuli in the Simon task, according to the study by Proctor and Lu (1999). The participants were asked to respond by pressing a key with their left or right index finger, regardless of the spatial information (**Figure 1B**). They performed eight practice trials prior to the execution of the task. In practice trials, feedback was provided regarding performance. Participants were also informed that responses had to be made within 500 ms and that the Japanese character indicating “too late” would be presented for 1000 ms whenever there was no response within the allowed time.

### Shooting Test

In both the pre- and post-tests, the participants shot toward a standard-sized lacrosse goal (1.83 m × 1.83 m) that was guarded by a goalie. The initial movement by the goalie was restricted to the horizontal dimension, and was based on an instruction (step left or step right) by the experimenter, of which each participant was unaware. After the initial movement (determined by the experimenter), the goalie could move freely to guard the goal. Each trial began with the cue provided by the experimenter; both the shooter and goalie were instructed to start their action as soon as possible after the cue. The participants performed 10 overhand shots standing 7 m away from the goal. Typical techniques used by goalies, such as a feint with the eyes or body direction, were prohibited as processing of the observed gaze, body, or head direction has been shown to impact the Simon effect (Hietanen, 2002; Ansorge, 2003; Pomianowska et al., 2011).

### Training Phase

A computer-based version of either the Type 2 task or the 2-back task was used for training. Participants underwent 10 sessions involving 180 trials each within four consecutive weeks. Eight practice trials were executed prior to performance of each task in every training session; during these practice trials, feedback was provided to ensure accurate mapping between the stimulus and response.

In the SRC group, the participants performed an SRC task with spatially incompatible mapping (Proctor and Lu, 1999). Participants were asked to respond using their left or right index fingers according to the spatial information. They had to press with their left finger for the right-side presentation of characters and vice versa within 500 ms, otherwise “too late!” written in Japanese was presented for 1000 ms (**Figure 1C**).

In the 2-back task, the participants had to respond with their right index finger whenever the current stimulus was identical to the previous stimulus, and with their left index finger when it did not match. Four types of characters (A, H, M, and S) were used (**Figure 1D**).

### Statistical Analysis

Both the reaction time and accuracy (% correct) were recorded in the computer-based training, and these were subjected to one-way analysis of variance (ANOVA) with repeated measures on the variable of the training session (1st, 2nd, …10th). In the cognitive test, the reaction time and accuracy of the Simon task were subjected to a mixed three-way ANOVA with one between-measure (group, SRC/2-back) and repeated measures on the variables of time (pre-/post-test) and compatibility (compatible/incompatible stimulus). Angular transformation was applied for the accuracy rate before performing ANOVAs. The Simon effect was calculated by subtracting the reaction time to the compatible stimulus from that of the incompatible stimulus, as described in previous studies (e.g., Rubichi et al., 1997; Tagliabue et al., 2000); a more positive value indicated a larger Simon effect. The score of the Simon effect and performance in lacrosse shooting were statistically analyzed using two-way ANOVA with one between measures (group) and one repeated measures (time) variable. Whenever *post hoc* tests were needed, paired-sample *t*-tests with a Bonferroni correction were used for pair-wise comparisons. When Mauchly’s test revealed a violation of the assumption of sphericity, corrected *p*-values were reported with uncorrected degrees of freedom and the Greenhouse-Geisser epsilon (ε). Partial eta squared ($\eta_{p}^{2}$) and Cohen’s *d* were reported to indicate the magnitude of effect sizes. Pearson correlations between the change in the magnitude of the Simon effect and the change in lacrosse shooting performance were calculated. A power analysis was carried out with G^∗^Power 3 (Faul et al., 2007). *Post hoc* analysis involving *F*-tests for within–between interaction was applied. In the power analysis, α was set at 0.05 and the correlation among repeated measures was set at 0.50.

## Results

### Training Phase

**FIGURE 2:**
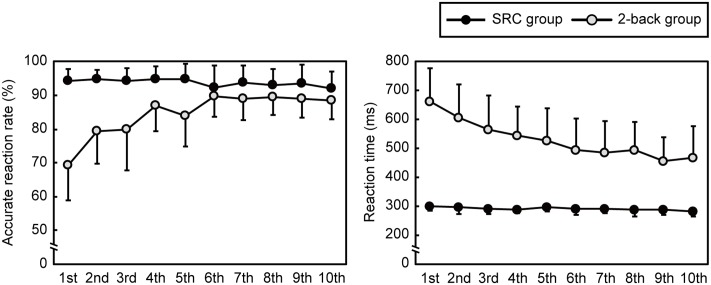
Participants’ performance in the Type 2 task (*n* = 15) and 2-back task (*n* = 14) during training sessions. The accurate response reaction rate is presented as a percent (Left), and the reaction time is presented in ms (Right). The error bars represent one standard deviation.

The performances in both the SRC and 2-back group during the training phase are illustrated in **Figure 2**. The data for two participants who had missing values in the 2-back group were excluded from the statistical analyses. The 2-back group showed a gradual increase in performance, but the SRC group did not because of a ceiling effect.

One-way ANOVA for accuracy indicated that the main effect in the SRC group reached marginal significance, *F*(9,126) = 1.9, *p* = 0.058, $\eta_{p}^{2}$ = 0.12, but there was no significant difference in the *post hoc* tests (*p*s > 0.05), while the main effect in the 2-back group was statistically significant, *F*(9,99) = 14.5, ε = 0.44, *p* < 0.0001, $\eta_{p}^{2}$ = 0.57. *Post hoc* comparisons showed increased accuracy during the 4th, 5th, 6th, 7th, 8th, 9th, and 10th sessions as compared with the 1st session; moreover, the accuracy during the 6th and 10th sessions was greater than that during the 2nd session, *t*s(11) ≥ 4.6, *p*s ≤ 0.037, *d*s ≥ 1.2.

One-way ANOVA for reaction time revealed a significant main effect in both the SRC and 2-back group, *F*(9,126) = 2.5, *p* = 0.011, $\eta_{p}^{2}$ = 0.15, *F*(9,99) = 12.1, ε = 0.40, *p* < 0.0001, $\eta_{p}^{2}$ = 0.52, respectively. *Post hoc* comparisons of the SRC group showed that the reaction time in the 10th session was shorter than that in the 5th session, *t*(14) = 4.1, *p* = 0.046, *d* = 0.9. Additionally, *post hoc* tests for the 2-back group showed that the reaction time in the 1st session was longer than that in the 4th, 7th, 8th, 9th, and 10th sessions; moreover, the reaction time in the 9th session was shorter than in the 2nd, 3rd, and 4th session, *t*s(11) ≥ 4.6, *p*s ≤ 0.036, *d*s ≥ 1.0.

### Cognitive Test (Simon Task)

**FIGURE 3:**
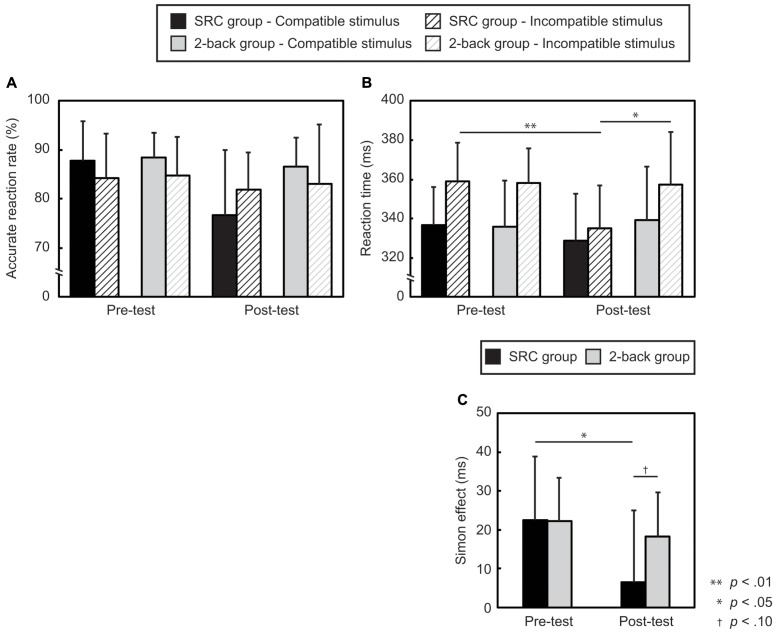
Participants’ performance during the Simon task. **(A)** The accurate response reaction rate (%) and **(B)** the reaction time are presented. **(C)** The Simon effect in both the SRC group and the 2-back group. The Simon effect was calculated by subtracting the reaction time to the compatible stimulus from the incompatible stimulus. The error bars represent one standard deviation.

**Figure 3** shows measures of performance during the Simon task. As expected, repetitive exposure to a Type 2 task significantly reduced the Simon effect; however, repetition of the 2-back task did not significantly change performance of the Simon task.

Three-way ANOVA for accuracy revealed a significant main effect of time, *F*(1,27) = 20.3, *p* = 0.0001, $\eta_{p}^{2}$ = 0.43, indicating that responses were more accurate in the pre- than in the post-test. There was also an interaction between group and time, *F*(1,27) = 8.6, *p* = 0.007, $\eta_{p}^{2}$ = 0.24. The SRC group responded more accurately in the pre- than in the post-test, *t*(14) = 5.6, *p* = 0.0001, *d* = 1.0. The responses of the SRC group were also less accurate than those of the 2-back group, in the post-test, *t*(27) = 2.1, *p* = 0.042, *d* = 0.8 (**Figure 3A**).

Three-way ANOVA for reaction time revealed a significant main effect of compatibility, *F*(1,27) = 60.7, *p* < 0.0001, $\eta_{p}^{2}$ = 0.69, and an interaction between time and compatibility, *F*(1,27) = 8.9, *p* = 0.006, $\eta_{p}^{2}$ = 0.25. Moreover, an interaction between group, time, and compatibility reached marginal significance, *F*(1,27) = 3.3, *p* = 0.08, $\eta_{p}^{2}$ = 0.11.

Because it has already been reported that repetition of the Type 2 task could affect reaction time in the Simon task, and because we had hypothesized *a priori* that there would be a significant difference between the groups, *post hoc* tests for the three-way interaction were conducted, and the result was marginally statistically significant. To decompose the three-way interaction, two-way ANOVAs were conducted for each level of each factor. A two-way ANOVA for Test × Compatibility yielded an interaction in the SRC group, *F*(1,14) = 8.1, *p* = 0.01, $\eta_{p}^{2}$ = 0.37, but not for the 2-back group, *F*(1,13) = 1.4, *p* = 0.27, $\eta_{p}^{2}$ = 0.09. In the SRC group, the Simon effect was prominent in the pre-, but not in the post-test, *t*(14) = 5.2, *p* = 0.0001, *d* = 1.1, *t*(14) = 1.3, *p* = 0.20, *d* = 0.3. The reaction time to the incompatible stimuli in the SRC group was reduced from pre- to post-test, *t*(14) = 3.6, *p* = 0.003, *d* = 1.1. Moreover, the two-way ANOVA for Group × Test for the incompatible stimuli revealed an interaction, *F*(1,27) = 5.7, *p* = 0.02, $\eta_{p}^{2}$ = 0.17. *Post hoc* tests showed that the SRC group responded faster to incompatible stimuli than did the 2-back group in the post-test, *t*(27) = 2.4, *p* = 0.02, *d* = 0.9. Moreover, the SRC group responded faster to incompatible stimuli in the post- than in the pre-test, *t*(14) = 3.6, *p* = 0.003, *d* = 1.1 (**Figure 3B**).

The Simon effect was defined as the difference in the reaction time for the incompatible vs. the compatible stimuli (incompatible reaction time-compatible reaction time). Two-way ANOVA for the Simon effect yielded a significant main effect of time, *F*(1,27) = 8.9, *p* = 0.006, $\eta_{p}^{2}$ = 0.25; a larger Simon effect was found in the pre- than in the post-test. The interaction between time and group reached marginal significance, *F*(1,27) = 3.3, *p* = 0.08, $\eta_{p}^{2}$ = 0.11. Despite this marginal significance, we conducted pair-wise comparisons to examine this interaction further, as we had *a priori* hypothesized that repetition of a Type 2 task would reduce the Simon effect, but that repetition of the 2-back task would not. Consistent with our prediction, *post hoc* comparisons revealed that the SRC group showed an attenuated Simon effect, *t*(14) = 2.8, *p* = 0.01, *d* = 0.9, while the 2-back group did not, *t*(13) = 1.2, *p* = 0.27, *d* = 0.3. The difference between the groups in the post-test reached marginal significance, *t*(27) = 2.0, *p* = 0.05, *d* = 0.8 (**Figure 3C**).

### Shooting Test

**FIGURE 4:**
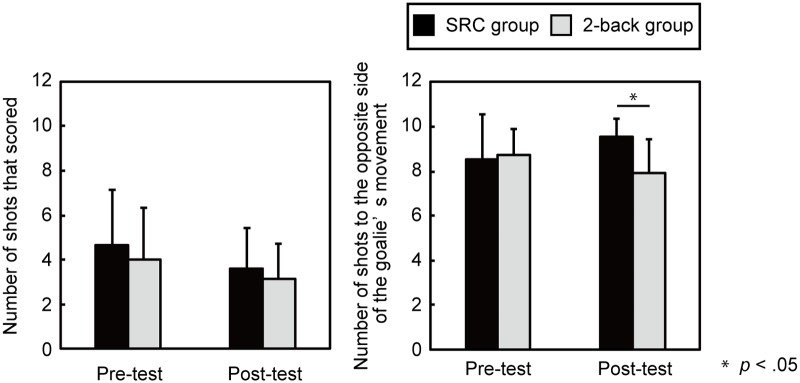
Participants’ performance in the lacrosse shooting test. The number of shots that scored points (Left) and the number of shots in the direction opposite of the goalie’s movement (Right) are depicted. The error bars represent one standard deviation.

**Figure 4** shows the pre- and post-test performance in terms of lacrosse shooting. The lacrosse shooting test was comprised of 10 shots. Performance was measured as the number of shots that scored or that were directed in the direction opposite to that of the goalie’s movement.

The number of shots that scored in both the pre-test and post-test was subjected to a two-way ANOVA. Both groups had a significant decrease in the number of successful shots, *F*(1,27) = 5.1, *p* = 0.03, $\eta_{p}^{2}$ = 0.16 (**Figure 4**, Left).

A two-way ANOVA for the number of shots toward the direction opposite of the goalie’s movement revealed an interaction between group and test, *F*(1,27) = 4.9, *p* = 0.04, $\eta_{p}^{2}$ = 0.15. A power analysis indicated that the statistical power of the interaction was high (1-β = 0.99).

*Post hoc* tests showed that the SRC group shot significantly more often in the direction opposite to that of the goalies’ movement than did the 2-back group in the post-test, *t*(27) = 3.6, *p* = 0.001, *d* = 1.3 (**Figure 4**, Right). The number of shots toward the direction opposite to the goalie’s movement was also subjected to a Mann-Whitney *U*-test (as the distribution of this data deviated from normal). The Mann-Whitney *U*-test revealed that, in the post-test attempts, the SRC group (*Mdn* = 10) shot in the direction opposite to the goalies’ first step more often than did the 2-back group (*Mdn* = 8) *U* = 40.5, *p* = 0.003. This finding supported the results of the two-way ANOVA and its *post hoc* comparisons.

### Relationship between the Simon Effect and the Number of Shots in the Direction Opposite to That of the Goalie’s Movement

**FIGURE 5:**
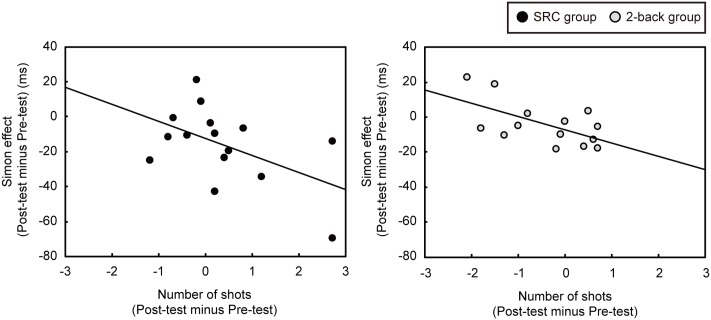
The relationship between the change in the magnitude of the Simon effect (post-test minus pre-test) and the difference in the number of shots in the direction opposite to that of the goalie’s movement (post-test minus pre-test). The negative correlations indicate that the more the players attenuated the magnitude of the Simon effect, the more their number of shots to the direction opposite of the goalie’s first step increased.

The Simon effect was calculated by subtracting the reaction time to the compatible stimulus from the reaction time to the incompatible stimulus (Tagliabue et al., 2000). **Figure 5** shows the relationship between the difference in the Simon effect (post-test minus pre-test) and the difference in the number of shots in the direction opposite to that of the goalie’s movement (post-test minus pre-test) in each group. There was a significant negative correlation in the 2-back group, *r* = -0.60, *p* = 0.02; moreover, the negative correlation reached marginal significance in the SRC group, *r* = -0.51, *p* = 0.05.

## Discussion

The main purpose of this study was to examine whether practice of a Type 2 task with incompatible spatial mapping could affect lacrosse shooting performance. We found that repetitive practice of the Type 2 task reduced the Simon effect, which changed the attention-allocation strategy in lacrosse shooting. Secondly, the magnitude of change in the Simon effect from pre- to post-test correlated negatively with the change in the number of shots toward the direction opposite to the goalie’s first movement.

During the training phase, neither reaction time nor accuracy improved for the SRC group, showing a floor/ceiling effect. This appears to be due to the simpler decision making required for the Type 2 task compared with the 2-back task that relies on memory retrieval. It is plausible that the participants fully acquired the incompatible spatial mapping during earlier stages of the training sessions with redundant execution of the Type 2 task that lasted even after the asymptote.

On the other hand, the 2-back group showed both a significant decrease in reaction time and an increase in the response accuracy in the 2-back task, indicating that they had enhanced their temporal-memory function (i.e., working memory). A previous study had confirmed plasticity in the brain region associated with working memory, including the middle frontal gyrus (Westerberg and Klingberg, 2007), during execution of a visuo-spatial working memory task. Although the 2-back group improved their memory performance, they did not acquire mapping of the horizontal spatial information between the stimulus and response location; the stimuli were consistently presented at the center of the monitor in the 2-back task.

When we compared the pre- versus post-test performance (i.e., reaction time and response accuracy) in the Simon task, the SRC group showed improvements in their performance (pre-test: 22 ms, post-test: 6 ms), whereas the 2-back group showed no significant change in their performance (pre-test: 22 ms and post-test: 18 ms). As we expected, the reaction time to incompatible stimuli was shortened in the SRC group, indicating that the Simon effect was significantly attenuated, but not reversed, by repetitive execution of a Type 2 task that involved incompatible spatial mapping.

It is possible that the incomplete reversal of the Simon effect could be ascribed to the age of the participants. Tagliabue et al. (2000) demonstrated a complete reversal of the Simon effect in children aged 5-8 years after repeated exposure to a spatial incompatibility task; on the other hand, young adult participants (aged 19-25 years) showed only attenuation of the Simon effect. Thus, it is reasonable to assume that the Simon effect cannot be reversed in young adults, because they have already consolidated the strong link between spatially corresponding stimuli and response locations. Thus, it is plausible that participants in our study (aged 19-23 years) had also acquired this strong link, which might have prevented them from experiencing a reversal of the Simon effect. It should be emphasized that the reaction time to a spatially incompatible stimulus was significantly shorter in the SRC group than in the 2-back group during the post-test, confirming attenuation of the Simon effect in the SRC group after completion of computer-based cognitive training.

Consistent with our hypothesis, the SRC group shot more frequently toward the opposite side of the goalie’s initial movement than the 2-back group. Among possible explanations, the attenuated Simon effect, induced by repetitive exposure to the Type 2 task, appears to be responsible for the increase in the number of shots to the opposite direction of the goalie’s movement. As the repetition of the Type 2 task created a short-term memory representation where the stimulus–response coding was reversed, participants increased shots to the opposite direction of the goalie’s movement. It has been reported that a reversal of the Simon effect can be caused by an attentional shift (Rubichi et al., 1997). Therefore, it is possible that the repeated execution of the Type 2 task enhanced attentional shift, which may have resulted in the increased shots to the opposite direction of the goalie’s movement.

Another possible explanation is that the repetition of the Type 2 task improved the capacity of the visual attentional system. It has been demonstrated that playing action video games enhances visual attention capacity (Green and Bavelier, 2003); however, it remains unclear what factor was important to achieve this. Given that the Type 2 task shares similar aspects with the action video game (e.g., having to process visuo-spatial information and respond to visual stimuli as quickly as possible), the repetition of the Type 2 task may also improve the visual attention capacity.

Contrary to our hypothesis, the number of successful goals in the post-test did not differ between the two groups. The ability to score was not improved by the repetitive exposure of the Type 2 task, although participants learned to allocate attention to the opposite side of the goalie’s movement. Given that the ball velocity is also important for scoring in lacrosse, the computer-based cognitive training alone is not sufficient and should be combined with physical training to improve ability to score. Future studies are needed to identify a proper protocol for the Type 2 training to increase scores. It has not previously been demonstrated that attenuation of the Simon effect could create representation of the non-corresponding stimulus–response association and could modulate an attentional shift that affects a dynamic response, such as lacrosse shooting.

We also expected that the change in magnitude of the Simon effect after repeated execution of the Type 2 task would correlate with an increased number of shots to the direction opposite of the goalie’s initial movement. Indeed, we found a significant negative correlation in the SRC group. However, contrary to our prediction, we also obtained a significant negative correlation for the 2-back group. Interestingly, the 2-back group showed an even higher *r*-value than that of the SRC group. These results may indicate that players who are better at attentional control, which is reflected in performance of the Simon task, may inherently have a superior shooting ability, independent of SRC training. On the other hand, SRC training enhanced the ability for attentional control, especially in players who had not acquired this ability (in the SRC group), resulting in a lower *r*-value in this group.

It should be noted that our study contains some limitations. First, the experimental schedule was not completely controlled. The training phase lasted more than 4 weeks, and the schedule for the computer-based training was adjusted to individuals’ availability. Thus, the inter-training interval differed among participants. Second, daily activities other than training were not controlled and monitored. Because all participants had a lacrosse practice schedule arranged by the university, the amount and the types of lacrosse practice did not appear to differ among the participants. Nevertheless, it cannot be ruled out that any other uncontrolled activities, including sports training and other video games, may have influenced shooting performance in this study. Third, there was a difference in a task difficulty between the Type 2 task and the 2-back task. There was a ceiling effect associated with the Type 2 task, but none for the 2-back task. Lastly, the goalie’s first step was determined by the experimenter and techniques to guard the goal were prohibited. Thus, the current study did not completely imitate a real game situation. However, our results showed that repetition of the Type 2 task can be a potential training tool for developing adequate attentional allocation in order to increase shooting performance. As far as we know, this is the first study to show changes in attention-allocation strategy in sports performance.

## Conclusion

Our results clearly showed that the attention-allocation strategy induced by the Type 2 training transferred to real lacrosse shooting performance. However, it still remains unclear as to whether the repetition of an SRC task directly enhances scoring ability in lacrosse. Attentional control is a crucial function for optimizing sports performance (Williams and Davids, 1998). Our findings suggest a potential training method, with a solid theoretical basis, for modulating the direction of attention allocation, particularly for novice players.

## Ethics Statement

This study was carried out in accordance with the recommendations of the Waseda University Ethics Committee with written informed consent from all subjects. The protocol was approved by the Waseda University Ethics Committee.

## Author Contributions

TH and HM contributed to the conception and design of the study. TH acquired and analyzed the data, and drafted the manuscript. HM critically revised the manuscript. Both authors approved the version of the manuscript to be published.

## Conflict of Interest Statement

The authors declare that the research was conducted in the absence of any commercial or financial relationships that could be construed as a potential conflict of interest.
